# Small Diameter (7.5 Fr) Single-Use Flexible Ureteroscopy with Direct In-Scope Suction (DISS^TM^) in Conjunction with Aspiration-Assisted Flexible Access Sheath: A New Hype for Real Stone-Free?

**DOI:** 10.3390/jcm13237191

**Published:** 2024-11-27

**Authors:** Petrișor Geavlete, Cristian Mareș, Răzvan Mulțescu, Dragoș Georgescu, Cosmin-Victor Ene, Valentin Iordache, Bogdan Geavlete

**Affiliations:** 1 Department of Urology, “Carol Davila” University of Medicine and Pharmacy, 050474 Bucharest, Romania; geavlete@gmail.com (P.G.); razvanmultescu@yahoo.com (R.M.); dgeorgescu2000@yahoo.com (D.G.); cosmin85_ene@yahoo.com (C.-V.E.); bogdan_geavlete@yahoo.com (B.G.); 2 Department of Urology, “Saint John” Clinical Emergency Hospital, 042122 Bucharest, Romania; pc_valii@yahoo.com; 3 Department of Urology, “Sanador Hospital”, 010992 Bucharest, Romania

**Keywords:** flexible ureteroscopy, single-use ureteroscopy, direct in-scope suction, DISS, suction ureteral access sheath

## Abstract

**Background**: Renal lithiasis continues to represent a great challenge for modern urology in terms of minimally invasive management of lithotripsy procedures. The recent revolution of endoscopes with the miniaturization of instruments and the development of improved disposable ureteroscopes combined with recent suction methods such as suction access sheaths or direct in-scope suction (DISS^TM^) systems promise to further improve the outcome of renal stone treatment. **Methods**: Considering this technological advance, this study aims to evaluate the results obtained by combining these methods in three groups: Group 1—Single-use 7.5 Fr flexible ureteroscope with standard access sheath, Group 2—Single-use 7.5 Fr flexible ureteroscope with direct in-scope suction (DISS^TM^) with standard access sheath, Group 3—Single-use 7.5 Fr flexible ureteroscope with DISS^TM^ with suction access sheath. A total number of 105 consecutive patients were enrolled in the study, divided equally in each group. Several parameters were followed, such as size and location of the stones, operative time, duration of hospitalization, the need for ureteral stent insertion, residual fragments, and subjective evaluation of the lithotripsy environment visualization. **Results**: The best results were highlighted in Group 3 for almost all evaluated parameters. Significant improvements were indicated in terms of stone-free rates. **Conclusions**: Additional large studies are needed to support these initial results, but preliminary data indicate a valuable advantage for every urologist who treats urolithiasis.

## 1. Introduction

Renal lithiasis, a prevalent urological condition, has been recognized since ancient times and has been thoroughly researched. It continues to be a significant concern for medical professionals, having a continuously increasing incidence from 4% in 1970 to 9% in 2010 and reaching over 10% in 2016 in the United States in the general population [1,2]. Moreover, the incidence of this condition is increasing in Europe. Germany reports a rise from 120 cases per 100,000 inhabitants in 1980 to over 700 cases per 100,000 inhabitants in the 2000s [3], while Italy reports 1.7 cases per 1000 inhabitants [4]. In certain regions, there has been a reported increase of over 37% in the past two decades [5,6]. If not detected and treated promptly, it may lead to various complications, including renal colic, hydronephrosis, urinary tract infections (UTI), pyelonephritis, pyonephrosis, and sepsis. The risk of chronic kidney disease (CKD) is being increasingly associated with nephrolithiasis, according to emerging evidence [7].

In the last 20 years, the modern treatment of renal lithiasis was represented by the classic triad—extracorporeal shock wave lithotripsy (ESWL), percutaneous nephrolithotomy (PCNL), and retrograde intrarenal surgery (RIRS) represented by flexible ureteroscopy. The European Association of Urology Guidelines on Urolithiasis (EAU) recommends flexible ureteroscopy as the first line treatment in kidney stones of medium size between 10 and 20 mm and as a reliable option in any type of kidney stones [8]. Through the increased technology and the improved performance of the new ureteroscopes, several authors claim the successful use of this treatment method for larger or staghorn stones [9,10,11]. With the development of advanced ureteroscopes, imaging systems, and auxiliary equipment, ureteroscopy has emerged as a core procedure in endourology. The new ureteroscopes enable surgeons to maintain optimal operating settings, including adequate irrigation flow, excellent sight, and the ability to insert additional tools using progressively lower-scope calibers. Miniaturization aims to strengthen the method’s efficacy while minimizing the difficulties associated with advancing the instrument to the upper urinary tract and reducing tissue damage produced by the ureteroscope in the urinary system [12].

Single-use flexible ureteroscopes (su-fURS), often known as ‘disposable’, have been commercially accessible in the last decade. Their creation primarily arose from efforts to minimize maintenance needs and the accompanying expenses and address durability concerns such as deflection loss during repeated use. Consequently, they have attracted growing interest and an expanding collection of research, indicating a novelty swiftly embraced by specialists [13,14]. The ureteral access sheaths have also undergone a significant improvement recently through miniaturization, mobility, and flexibility, as well as the possibility of direct suction on the sheath, leading to a considerable increase in intraoperative visualization, reduction in operative time and, perhaps most importantly, suction of residual stones resulting from fragmentation in reaching the absolute desired result—real stone-free status [15,16,17,18]. Another important success of the recent technique is the fusion between the miniaturization of the ureteroscope and the advantage of suction in endourology by creating a reliable, small instrument that has the possibility of direct suction through the ureteroscope—direct in-scope suction (DISS^TM^), represented by the new ureteroscope Pusen^TM^ PU3033 -AH, with an external diameter of only 7.5 Fr and a working channel of 3.6 Fr [19].

The combination of the latest generation of flexible ureteroscope with suction power through the instrument, in combination with the flexible access sheaths used with or without suction, represents the latest technology in RIRS for kidney stones; the advantage of using these modern techniques is the absolute desire of the modern urologist in achieving the real stone-free status for the complete treatment of renal lithiasis. This paper aims to analyze the outcomes achieved through the combination of these devices and modern auxiliary instruments in managing renal lithiasis and to evaluate the features of these instruments, the intraoperative benefits they provide, and the results obtained in comparison.

## 2. Materials and Methods

A prospective study was conducted over a period of 5 months between 1 March and 31 July 2024 at the St. John Clinical Emergency Hospital in Bucharest, Romania. It followed 105 consecutive cases of renal lithiasis of hospitalized patients in the Department of Urology during this period when the necessary endourological instruments were available for use. Patients over 18 years old, with single intrarenal stones measuring up to 25 mm, which were not staghorn stones, and patients who did not present malformations of the upper urinary tract represented the inclusion criteria in this study. Patients with high-volume or staghorn stones or those with malformations of the upper urinary tract represented the exclusion criteria in the study. All patients evaluated by each method signed the informed consent at admission. The present study was conducted in accordance with the Declaration of Helsinki. It was approved by the hospital’s ethics committee (no 11752/17 February 2024).

### 2.1. Data Collected

General demographic data such as sex, age, and medical and surgical history were extracted from the observation files. Data on the location, size, and volume of the stones and the density measured in Hounsfield units (HU) were also noted. Intraoperatively, the characteristics of maneuverability and visualization in different situations of the devices were determined, and the operating time, as well as visualization determined subjectively by the experienced urologist who performed all the procedures, were noted. Postoperatively, the complications that occurred, as well as the hospitalization time, were determined. In all cases, kidney/ureter/bladder radiography or kidney ultrasound was performed three weeks postoperatively to evaluate the stone-free status, according to the recommendations of the EAU guidelines on Urolithiasis [8]. If a residual kidney stone was suspected, a non-enhanced CT scan was performed for evaluation. The computer programs IBM SPSS Statistics for Windows version 26.0 (IBM Corp. Released 2015. Armonk, NY, USA: IBM Corp) and Microsoft Excel Data Analysis (Microsoft 365 Personal) were used for systematization, grouping, processing, and statistical analysis operations of the information collected.

### 2.2. Study Design

**Figure 1:**
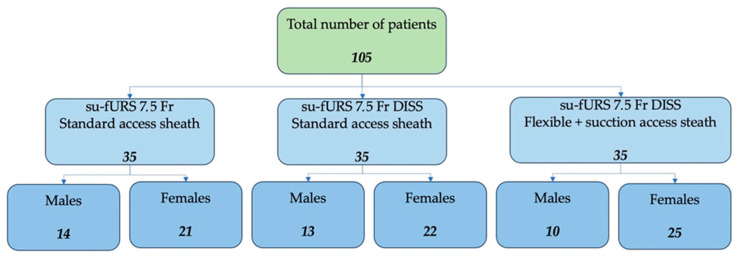
Flowchart representing the division of patients by sex, depending on the procedure and technique used.

A prospective study was conducted that followed 105 consecutive patients treated by RIRS using different modern technical methods, as follows: Group 1—in 35 patients, the single-use flexible 7.5 Fr ureteroscope was used, without aspiration, using the conventional ureteral access sheath (10/12 Fr); Group 2—in 35 patients we used the 7.5 Fr single-use flexible ureteroscope with direct in-scope suction (DISS^TM^) together with the conventional access sheath (10/12 Fr); Group 3—in 35 patients, the 7.5 Fr ureteroscope with direct in-scope suction (DISS^TM^) was used together with the flexible ureteral access sheath with suction—Clear Petra ^TM^ (Wellead, Guangzhou, China) or YiGaoMed ^TM^ Elephant II (YiGao Med, Hangzhou, China) 10/12 Fr. The distribution of the studied population according to the performed procedure is represented in Figure 1.

### 2.3. Technique and Devices

**Figure 2:**
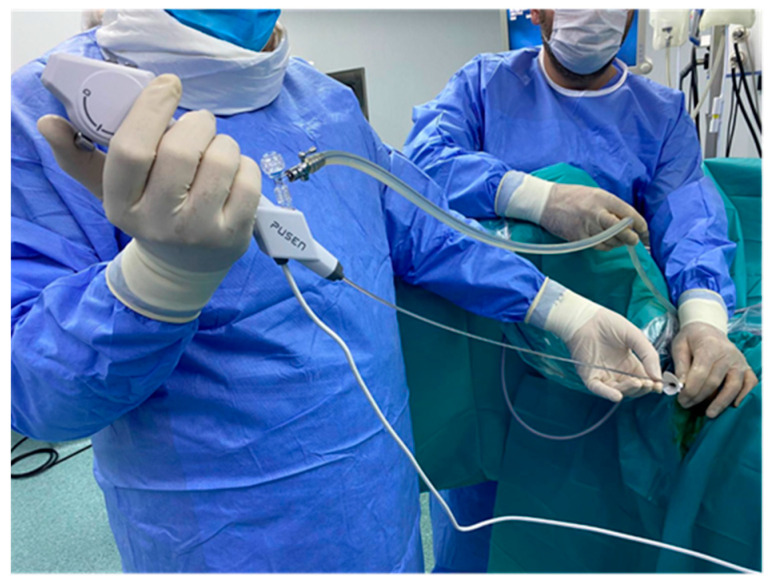
Small diameter 7.5 Fr single-use flexible ureteroscope Pusen PU 3033A with standard access sheath.

**Figure 3:**
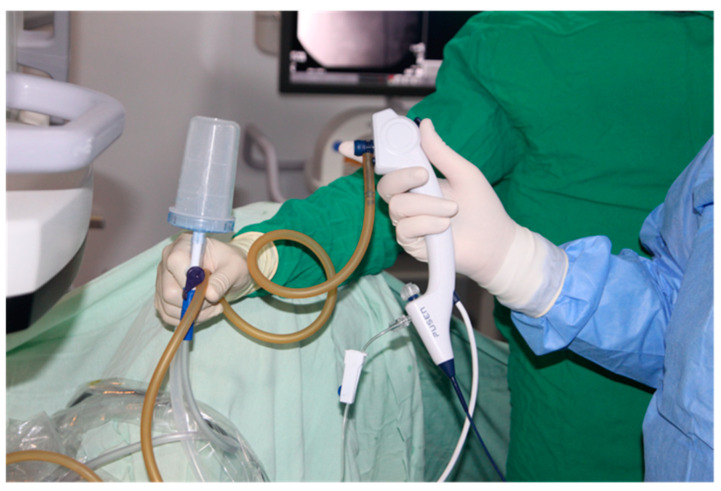
Small diameter 7.5 Fr single-use flexible ureteroscope Pusen PU 3033A with direct in-scope suction DISS TM with standard access sheath.

**Figure 4:**
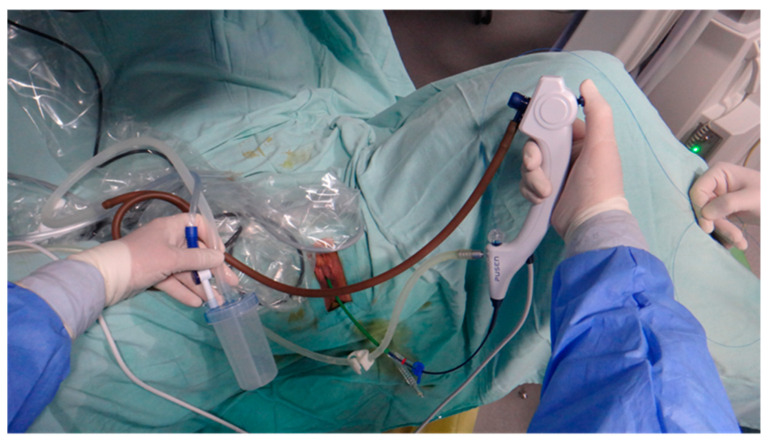
Small diameter 7.5 Fr single-use flexible ureteroscope Pusen PU 3033A with direct in-scope suction DISS TM with Clear Petra TM suction access sheath.

All procedures were performed by the same experienced urologist, considered an expert in the field, with over 20 years of experience in flexible ureteroscopy, consitantly performing over 1500 procedures annually. In all cases, spinal anesthesia was practiced with the patient placed in the standard lithotomy position. Following the use of cystoscopy to locate the openings of the ureters, a stiff hydrophilic guidewire with a diameter of 0.035 inches is inserted into the renal cavities with fluoroscopy guidance. The evaluation of the ureter is always performed under direct endoscopic visualization before advancing the ureteral sheath. Depending on the group of studied patients, a specific ureteral sheath is inserted, more precisely in groups 1 and 2, a conventional ureteral access sheath, and in group 3, a flexible ureteral access sheath with suction. Afterward, the pelvic-calyceal cavities are inspected to identify the stones. In group 1, the single-use flexible ureteroscope Pusen PU3033A 7.5Fr is used (Pusen Medical Technology Co., Zhuhai, Guangdong, China), and in groups 2 and 3, the single-use flexible ureteroscope Pusen PU3033AH-Standard 7.5Fr with the DISS^TM^ system is used. The set-up from group 1 (Pusen PU3033A 7.5 Fr scope with standard access sheath 10/12 Fr) is represented in Figure 2. The set-up from group 2 (Pusen PU3033A 7.5 Fr scope with direct in-scope suction DISS ^TM^ with standard access sheath 10/12 Fr) is represented in Figure 3. The set-up from group 3 (Pusen PU3033A 7.5 Fr scope with direct in-scope suction DISS ^TM^ and Clear Petra ^TM^ or YiGaoMed ^TM^ suction access sheath) is represented in Figure 4.

**Figure 5:**
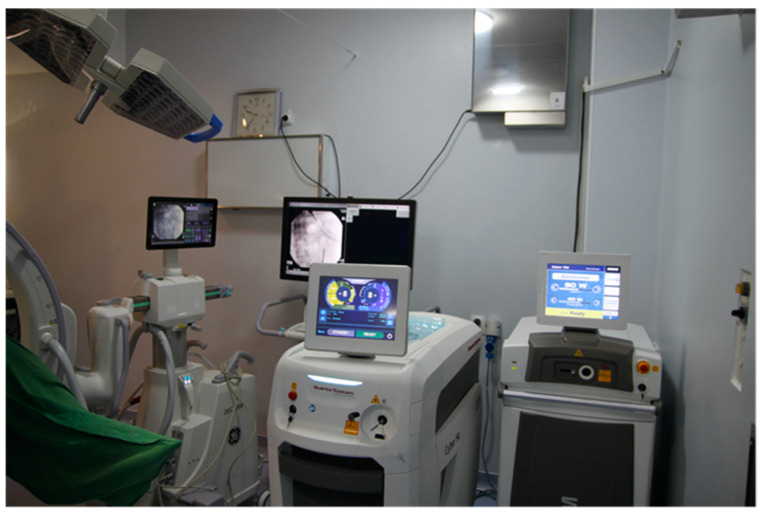
Laser System TFL (Thulium fiber laser) 60 W (Quanta System).

After identifying the stone, Thulium fiber laser (TFL—Quanta System, Milan, Italy) laser lithotripsy was performed using different settings (dusting or fragmenting). In most cases, the preferred setting was 0.4 J with 20 Hz (8 W energy), as shown in Figure 5. When required, baskets were used to retrieve stones. In terms of irrigation power, we aimed to maintain an average intracavitary pressure between 35 and 45 mm H_2_O. In this way, the visibility is optimal, but the risk of hyperpressure in the pyelocalyceal system is minimal. For aspiration, we used a vacuum power that varied between 85 and 150 mmHg, depending on the need, and for the fine adjustment, we used the access sheath valve. When a suction ureteral access sheath was used, it was positioned at the pyeloureteral junction level during stone lithotripsy for optimal maintenance of the irrigation flow and to increase visibility. However, when aspiration of the small residual fragments was necessary, the sheath was advanced together with the ureteroscope at the level of the calyx where the stone fragments resulting from lithotripsy were located. When stone fragments entered the sheath during suction, the ureteroscope was retracted into the sheath to facilitate their extraction into the specially designed reservoir. After the surgery, all calyceal cavities were inspected again to evaluate the immediate postoperative stone-free status. The urologist who performed the surgical procedure was assigned to analyze the visualization during the evaluation of all procedures. This was done to subjectively evaluate the clarity of the environment during the procedure using different ureteroscopes and stiff or bendable access sheath, with or without suction. When necessary, a JJ stent was inserted at the end of the procedure.

## 3. Results

A total number of 105 patients, 37 males, representing 35.23%, and 68 females, representing 64.76%, were prospectively evaluated for the RIRS treatment of pelvic-calyceal lithiasis by the previously described methods. The male patients’ ages ranged from 27 to 72 years, averaging 49.62 years. The female patients’ ages ranged from 22 to 67 years, with an average age of 44.6 years. In most cases, the location of the stones was at the level of the renal pelvis—43 cases (40.95%), followed by the location at the level of the lower calyx—32 cases (30.47%), and the middle calyx—24 cases (22.85%); the location of the calculus represented the fewest cases at the level of the upper calyx—6 cases (5.71%). A complete representation of the location of the stones according to the group of evaluated patients is represented in Table 1.

In all cases, stones under 25 mm were addressed, considered standard RIRS treatment of intrarenal stones by the European Association of Urology (EAU). The length of the stones varied between a minimum of 12 mm and a maximum of 25 mm, with an average of 18.84 mm. The width of the stones varied between 5 mm and 13 mm, with an average of 9.46 mm, while the height of the stones varied between 4 and 11 mm, with an average of 7.21 mm. More important than the maximum size of the stones is their volume, which was always calculated, varying between 160 mm^3^ and 1500 mm^3^, with an average of 672.71 mm^3^. A representation of all stone dimensions, volumes, and densities in the studied groups is represented in Table 2.

In each case, the operative time was measured from the first urethral access to the insertion of the urethral catheter at the end; in the first group, the average operative time was 50.28 min, with maximum durations between 30 and 90 min. In the second group, the average operative time was 53 min, with maximum durations between 35 and 80 min. In the third group, the average operative time was 52.5 min, with a maximum between 30 and 75 min. In terms of postoperative complications, in no case were severe complications that required reinterventions or advanced treatments, including intensive care therapy, encountered. All complications that occurred were reported as Clavien I and II. In the first group, hematuria was detected in 3 cases and fever in 2 cases. In the second group, hematuria was detected in 4 cases and fever in one case, while in the third group, only mild hematuria was detected in 2 cases. In all cases of fever, the patient’s hospitalization was extended by another night for antibiotic treatment and monitoring, while in cases of minimal hematuria on the urethral catheter on the first postoperative day, the patient was discharged with specific treatment at home. All uncomplicated ureteroscopy cases in our clinic are discharged on the first postoperative day. In cases with complications, the patient is kept hospitalized until they are completely resolved or out of danger of being discharged with treatment at home and re-evaluation recommendations. In the studied groups, only the patients who presented mild fever were hospitalized for an additional night for conservative treatment. In contrast, all the others were discharged on the first postoperative day. All patients in the third group were hospitalized for only one night. In all cases, when it is justified, a double J stent must be placed at the end of the retrograde ureteroscopy. In group I, in the case of 9 patients, a stent was placed (25.71%); in the second group, in the case of 8 patients, a double stent was placed Postoperative J (22.85%), while in the third group, only in 2 cases a double J stent was placed postoperatively (5.71%). The difference was statistically significant between groups I and III (*p* = 0.024).

The evaluation of the postoperative stone-free status was performed in all cases 3 weeks after the date of the procedures by renal ultrasound or kidney ureteral bladder radiography (KUB). Residual fragments are considered significant if they are larger than 3 mm; in cases where residual fragments are detected, non-enhanced computer tomography is performed to evaluate them and determine the further therapeutic protocol. In Group I, 6 patients, representing 17.14%, were diagnosed with residual stones, while in Group II, 4 cases with residual fragments were determined, representing 11.42%. Only one case with residual fragments was determined in Group III, representing 2.85% of all patients. The difference was statistically significant between groups I and III (*p* = 0.023). All cases were performed by a single senior urologist with over 30 years of experience in retrograde ureteroscopy. He gave a subjective grade regarding endoscopic visualization (image clarity, presence or absence/aspiration of dust resulting from stone fragmentation, presence or absence of small fragments resulting from lithotripsy, as well as the need to increase or decrease the level of irrigation, extraction of fragments with a basket probe) during lithotripsy, as well as overall experience regarding each group. He evaluated Group I with a grade of 6/10, Group II with a grade of 8/10, and Group III with a grade of 9/10. A complete evaluation of all operative and postoperative results is presented in Table 3.

## 4. Discussion

Flexible ureteroscopy has seen an impressive technological advance in the last decade, with the appearance on the market of multiple models of ureteroscopes from different manufacturers, with increasingly better technical characteristics, miniaturized instruments that offer a pleasant experience to the urologist who uses them, having at the same time improved results in terms of operating time, the success of lithotripsy which is no longer absolutely dependent on the size of the stone, postoperative complications, and stone-free rates. At the same time, single-use instruments have revolutionized the market of modern kidney stone treatment, bringing disposable, relatively cheap products with almost similar performance, if not even better in some cases, compared to standard, reusable devices. With the advent of single-use miniaturized ureteroscopes of 7.5 Fr, together with access sheaths with suction, postoperative results are improved, representing valuable tools in the armamentarium of any urologist who treats kidney stones.

### 4.1. The Advantages of Small Diameter Single-Use Flexible Ureteroscopy

With the miniaturization of endoscopes, there was a revolution in the endoscopic treatment of renal lithiasis, achieving easy access to stones regardless of their location at the pyelo-calyceal level while offering increased maneuverability. Single-use ureteroscopes provide an additional advantage in terms of intraoperative management of endoscope maneuverability without fear of damage during complex access maneuvers in hard-to-reach calyces. An objective ex vivo comparison of the newly available single-use endoscopes published last year demonstrated outstanding characteristics of these instruments in terms of visualization and color representation, image resolution, excellent deflection of the 7.5 Fr ureteroscope, as well as an irrigation flow comparable between 7.5 Fr and 9.5 Fr ureteroscopes [20]. The safety and efficiency of these 7.5 Fr endoscopes were recently successfully tested in the pediatric population in two large hospitals in Spain and the UK in a pilot study published this year [21]. The exceptional results were represented by the intraoperative success of accessibility of the stones regardless of their position, with a zero rate of postoperative complications and a stone-free rate of over 92%. Although the study was conducted on a small group of patients (26 children), the preliminary results opened new horizons for using small single-use endoscopes regardless of the patient’s characteristics. In an interesting comparison made by Shashank Agrawal et al. and published in the World Journal of Urology in 2021, the Pusen PU3033A 7.5 Fr single-use ureteroscope was compared with the Pusen PU3022A 9.5 Fr in terms of visualization, deflection, and maneuverability in a prospective study [22]. The outstanding results of the small-sized endoscope make it comparable to the 9.5 Fr endoscope in all the chapters studied, with the additional advantage of the reduced diameter that offers easier access and a favorable approach to stones in the upper urinary tract. Another interesting study that followed flexible ureteroscopy using the “no touch” technique published in 2021 compared the results of retrograde ureteroscopy without a sheath and ureteral access, using both reusable ureteroscopes and single-use endoscopes from Pusen—PU 3033A (7.5 Fr) and PU 3022A (9.5Fr). The best results were obtained with the small ureteroscope, optimizing the success rate and having the lowest complications [23]. In a cost-effective comparison published by Eugenio Ventimiglia et al. in which the problem of the high costs of using single-use endoscopes was raised, it was demonstrated that if we take into account all the costs of using reusable endoscopes, such as the purchase cost divided by the number of uses, sterilization, packaging, handling, repair, and warranty in comparison with the device that is disposable after a single use, it was found that the economic advantage must be taken into account in choosing the endoscope with which kidney stones are treated [24]. Considering both technical specifications that translate into increased efficiency and maneuverability as well as the cost-effectiveness ratio that is utterly important in modern medicine, the current directions of minimally invasive endoscopic surgery of the upper urinary tract are increasingly turning to disposable endoscopes that offer modern, extremely reliable, miniaturized devices that can be used without fear of failure and the need for repair.

### 4.2. Aspiration Through the Endoscope, Between Myth and Reality

The necessity of suctioning residual fine fragments during lithotripsy was a desire created since the first ureteroscopies, considering the improvement of visualization of the intrarenal environment for increased efficiency and reduction of operative time. An innovative method recently developed by several manufacturers is suction through the endoscope—direct in-scope suction (DISS^TM^), which connects an external suction system directly to the ureteroscope and applies a suction force through the lumen of the endoscope aspiring the fine fragments resulting from lithotripsy [25]. A study recently published in the Journal of Clinical Medicine by Vineet Gauhar et al. [26] followed two series of patients who underwent DISS ureteroscopies or ureteroscopies with suction access sheaths. In both cases, the results were favorable, both being feasible techniques for daily lithotripsy; however, some important elements were noted in the DISS group. First of all, it should be noted that the improved intraoperative visualization favors the approach of larger stones and, at the same time, regardless of their location at the level of the pyelo-calyceal system. This is comparable to the present study group in which calculi with a larger lithiasis volume and more challenging locations (inferior calyx) were approached compared to the group in which ureteroscopy without aspiration was used. On the other hand, the authors noted an additional operative time regarding the aspiration of fine dusting, something also pointed out in the present study. They emphasized the procedure’s usefulness and the feasibility of using it on a large scale to improve the RIRS technique.

Another very interesting study published in the spring of this year [27] demonstrated this procedure’s usefulness in a high-complexity case: antegrade ureteroscopy on the transplanted kidney for obstructive ureteral lithiasis. They used the aspiration technique through the ureteroscope, with successful lithotripsy of a large stone at the ureteral level. The high visualization of the lithotripsy environment was noted, a fact comparable to the results of the current study, in which the visualization of the lithotripsy environment was much higher when the suction system through the ureteroscope was used, compared to the group in which the standard ureteroscope was used without suction. Another review of the literature published this year in the Journal of Clinical Medicine [16] that followed the current methods of aspiration in RIRS available on the market showed the increased usefulness of the endoscope aspiration system noted by several authors, being almost unanimously accepted the idea of improvement visualization of the intrarenal environment during lithotripsy, a similar result to the present study.

### 4.3. Combining Suction Systems in RIRS for Improved Results

The role of suction in RIRS associated with lithotripsy of urinary stones is proven to have an increased efficiency with clear advantages, coming to the aid of the urologist who performs this procedure, but also to the benefit of the patient in the results obtained. By combining the current suction techniques through both the ureteroscope sheath and direct in-scope suction, at least on a theoretical level, the results should be improved. This assumption was presented in a systematic review published two months ago in BJUI Compass by Lazaros Tzelves et al. [28], in which the recent data from the literature about the role of suction in RIRS were identified; 16 recent studies were evaluated, of which 5 were randomized and 11 observational, concluding that the use of suction using ureteral access sheaths, ureteral catheters, or scopes has the potential to enhance stone-free rates (SFRs), decrease rates of both overall and infectious complications, and shorten the duration of hospitalization. The same result was observed in the groups studied in the present study; an improvement was observed both in terms of the need to insert the postoperative ureteral stent, the number of cases with residual stone fragments, the decrease in the duration of hospitalization, all cases in which they were used endoscope suction combined with suction sheath being managed as 24h hospitalization, as well as the subjective improvement of endoscopic visualization during stone fragmentation.

Another helpful method for improving results in RIRS is using predictability scores that consider several key elements of cases to suggest postoperative success. Such a score is the Resorlu–Unsal score, which was described for the first time in 2012 [29] and which evaluates multiple parameters such as stone size > 20 mm, lower calyceal stones, and infundibulo-pelvic angle < 45°, stone number > 1, and abnormal anatomy. It was used in comparison with other scores, such as the modified Seoul National University Renal Complexity (S-ReSC) score, Ito’s score, and S.T.O.N.E. score, demonstrating its usefulness in predicting the postoperative results starting from the clinical data and the stone characteristics from the preoperative evaluations [30]. Recently, in 2022 [31], it also proved its effectiveness through external validation, demonstrating once again the usefulness of prediction scores in general and that it is an easy-to-use grading system that has the potential to accurately and very efficiently estimate the postoperative stone-free rate after RIRS. A literature review published last year by Catalina Solano et al. [32], which sought to present the current results of retrograde ureteroscopy in which various intraoperative suction techniques are used, showed how ubiquitous the opinion of experts in the field regarding the combination of these techniques, having favorable results in various key factors related to the favorable outcome of intervention, such as controlling intrarenal pressure, improving postoperative stone-free rates and improving intraoperative visualization due to the dusting resulting from the fragmentation of urinary stones. The outcomes are significantly better regarding operating time reduction, global complication rate reduction, a finding also highlighted in this study, and infection rate reduction even when the ureteral access sheath with aspiration is the only suction method used in RIRS [33]. Additionally, in the current study, none of the patients in whom the suction sheath was used experienced postoperative septic phenomena, which implicitly led to a decrease in the duration of hospitalization. A large recently published study in August 2024 [34] conducted international, multicenter, and randomized trials in 8 large medical centers in China, the Philippines, Malaysia, and Turkey, following in comparative terms the standard ureteral access sheaths and the new bendable-type ureteral access sheaths showed that the latter performed better, with greater SFR, a lower postoperative fever rate, and better quality of life outcomes. It concluded that the new sheaths are a viable alternative in the treatment of renal lithiasis, offering clear advantages and reducing the risk of complications associated with the procedure. This fact was also emphasized in the results of the current study, in terms of stone-free rate, postoperative complications, length of hospitalization, as well as the improvement in intraoperative visualization during lithotripsy. Another paper published in March this year by Lujia Wang et al. [35], followed by a comparison of the results obtained in RIRS between the conventional, standard access sheath, and the suction access sheath, emphasized notable advantages in terms of postoperative stone-free rates, as well as the complications that occurred, especially the infectious ones, a fact emphasized by the results of the present study. Moreover, the authors recommend using these new sheaths with suction, especially in cases where postoperative septic complications are suspected. This again shows their high utility, especially since septic complications after RIRS can sometimes be redundant.

### 4.4. Limitations

The present study presents some limitations that must be discussed. First, the small group of patients in each group is an important limitation in expressing a clear conclusion regarding the differences between the three methods of performing the procedure. However, consecutive patients with an indication of RIRS were enrolled in the study, and the only selection that was considered was for the use of aspiration methods in patients with a larger lithiasis volume or, sometimes, when there were cases with stones positioned in hard-to-reach places, such as the lower calyx. Another important limitation that must be mentioned is that the study is unicentric and that all procedures were performed by a single urologist experienced in RIRS. Still, the latter consideration was useful precisely because of the attempt to standardize the results without the risk of bias between different experience for different operators. A third significant limitation of the current study is represented by the performance of all interventions with endoscopes from the same manufacturer without being able to compare the method itself. This fact was followed precisely out of the desire to standardize the results without making differences between different devices. A last limitation of this study is represented by the lack of information about the chemical composition of the stones so that no differences can be made according to the difficulty of their lithotripsy.

## 5. Conclusions

Endoscopic lithotripsy of kidney stones has revolutionized the minimally invasive management of urinary lithiasis. The last decade brought remarkable improvements regarding the miniaturization of instruments, the appearance of single-use endoscopes, and the development of suction methods for residual fragments. Widespread use of the new single-use ureteroscopes with a direct suction system through the scope combined with the new suction access sheaths can significantly improve stone-free rates. Large, randomized, multicenter studies are now needed to standardize the results and draw definite conclusions regarding the benefit of these procedures.

## Figures and Tables

**Table 1:** Evaluation of the location of kidney stones in each studied group.

	Group 1		Group 2		Group 3		Total	
	*n*	%	*n*	%	*n*	%	*n*	%
Renal pelvis	15	14.28	16	15.23	12	11.42	43	40.95
Inferior calix	9	8.57	9	8.57	14	13.33	32	30.47
Middle calix	10	9.52	7	6.66	7	6.66	24	22.85
Superior calix	1	0.95	3	2.85	2	1.9	6	5.71

**Table 2:** Evaluation of the dimensions, volumes, and stone density in the studied groups.

	Group 1			Group 2			Group 3			Total		
	Min	Max	Average	Min	Max	Average	Min	Max	Average	Min	Max	Average
Length (mm)	12	25	18.28	15	25	19.11	15	25	19.14	12	25	18.84
Width (mm)	5	12	8.94	5	12	9.57	6	13	9.88	5	13	9.46
Height (mm)	4	11	6.82	4	11	6.88	5	11	7.94	4	11	7.21
Volume (mm^3^)	160	1500	608.87	237.5	1254	645	270	1391.5	763.38	160	1500	672.71
Stone density (HU)	450	1380	956	460	1350	1017.42	460	1380	1000.85	450	1380	991.42

**Table 3:** Evaluation of the results in terms of operative time, complications, hospitalization time, postoperative stent, residual stones, and subjective analysis of visualization during lithotripsy.

	Group 1			Group 2			Group 3		
Operative Time (min)	Min	Max	Average	Min	Max	Average	Min	Max	Average
	30	90	50.28	35	80	53	30	75	52.5
Complications	Mild Hematuria—3 cases Postoperative Fever—2 cases			Mild Hematuria—4 cases Postoperative Fever—1 case			Mild Hematuria—2 cases		
Hospital time (days-average)	1.085			1.057			1		
Postoperative ureteral stent Number of cases (percentage)	9 (25.71)			8 (22.85)			2 (5.71)		
Residual stones Number of cases (percentage)	6 (17.14)			4 (11.42)			1 (2.85)		
Subjective analysis of visualization	6/10			8/10			9/10		

## Data Availability

The data presented in this study are available on request from the corresponding author due to the technical limitation concerning the external access to the server where they are deposed.
